# Determining eligibility for antiretroviral therapy in resource-limited settings using total lymphocyte counts, hemoglobin and body mass index

**DOI:** 10.1186/1742-6405-4-1

**Published:** 2007-01-18

**Authors:** David M Moore, Anna Awor, Robert S Downing, Willy Were, Peter Solberg, David Tu, Keith Chan, Robert S Hogg, Jonathan Mermin

**Affiliations:** 1 Global AIDS Program, US Centers for Disease Control and Prevention, Entebbe, Uganda; 2 British Columbia Centre for Excellence in HIV/AIDS, Vancouver, BC, Canada; 3 Department of Medicine, Faculty of Medicine, University of British Columbia, Vancouver, BC, Canada; 4 Institute for Global Health, University of California, San Francisco, San Francisco, California; 5 Medicins Sans Frontieres – Holland, Amsterdam, The Netherlands

## Abstract

**Background:**

CD4+ T lymphocyte (CD4) cell count testing is the standard method for determining eligibility for antiretroviral therapy (ART), but is not widely available in sub-Saharan Africa. Total lymphocyte counts (TLCs) have not proven sufficiently accurate in identifying subjects with low CD4 counts. We developed clinical algorithms using TLCs, hemoglobin (Hb), and body mass index (BMI) to identify patients who require ART.

**Methods:**

We conducted a cross-sectional study of HIV-infected adults in Uganda, who presented for assessment for ART-eligibility with WHO clinical stages I, II or III. Two by two tables were constructed to examine TLC thresholds, which maximized sensitivity for CD4 cell counts ≤ 200 cells μL, while minimizing the number offered ART with counts > 350 cells μL. Hb and BMI values were then examined to try to improve model performance.

**Results:**

1787 subjects were available for analysis. Median CD4 cell counts and TLCs, were 239 cells/μL and 1830 cells/μL, respectively. Offering ART to all subjects with a TLCs ≤ 2250 cells/μL produced a sensitivity of 0.88 and a false positive ratio of 0.21. Algorithms that treated all patients with a TLC <2000 cells/μL, excluded all patients with a TLC >3000 cells/μL, and used Hb and/or BMI values to determine eligibility for those with TLC values between 2000 and 3000 cells/μL, marginally improved accuracy.

**Conclusion:**

TLCs appear useful in predicting who would be eligible for ART based on CD4 cell count criteria. Hb and BMI values may be useful in prioritizing patients for ART, but did not improve model accuracy.

## Background

Guidelines developed by the World Health Organization (WHO) for the use of antiretroviral therapy (ART) in low-income countries state that HIV-infected individuals should commence ART if they have WHO stage IV disease, stage III disease and a CD4+ T lymphocyte (CD4) cell count of ≤350 cells/μL, or stage I or II disease with CD4 cell counts ≤200 cells/μL [1]. Recently WHO has recommended to increase this threshold for stage I and II individuals to 350 cells/μL [2].

If CD4 cell counting is not available, as is the case in most of sub-Saharan Africa, the WHO guidelines recommend using clinical staging alone, or in combination with total lymphocyte counts (TLCs) of < 1200/μL in order to determine ART eligibility[1]. However, many studies have found both clinical stages III/IV and this TLC threshold to have poor sensitivity for low CD4 cell counts, leading researchers attempt to define other TLC thresholds which better correspond to CD4 cell counts ≤ 200 or 350 cells/μL [3-5]. Studies which have incorporated hemoglobin (Hb) or hematocrit into clinical algorithms have shown improved performance of TLC in predicting low CD4 cell counts[6-8]. However, such studies have treated the CD4 cell count threshold as an absolute standard, where initiating treatment in patients with cell counts above 200 cells/μL is undesirable. As delaying ART until CD4 cell counts fall below 200 cells/μL results in increased mortality[9] and most studies from sub-Saharan African settings have shown high mortality rates in the first year on therapy [10-12], it seems reasonable to adopt the more liberal ART eligibility criteria of <350 cells/μL.

We designed an analysis among HIV-infected adults in rural Uganda being screened for ART eligibility. We examined the clinical utility of TLCs, Hb, and body mass index (BMI) to maximize the sensitivity to detect individuals with CD4 cell counts below 200 cells/μL, and limit the proportion of individuals who would be offered ART with CD4 cell counts above 350 cells/μL.

## Methods

The Home-Based AIDS Care Program (HBAC) is a clinical trial of three different monitoring strategies for patients receiving ART in rural Eastern Uganda. Registered clients of The AIDS Support Organization (TASO), a local HIV/AIDS care and support organization in Tororo and Busia districts, were invited to be screened for ART eligibility. The study includes participants from a prior diarrhea prevention and cotrimoxazole study described elsewhere [13], as well as newly recruited clients. Aggregated data from screening at baseline were used for this analysis. The studies were approved by the Science and Ethics Committee of the Uganda Virus Research Institute and the Institutional Review Boards of the Centers for Disease Control and Prevention and the University of California, San Francisco.

All subjects in this analysis were HIV-infected adults aged ≥ 18 years. Clinical and laboratory assessments at baseline included a complete blood counts (CBC), viral load, and CD4 cell counts. Those who had a CD4 count ≤250 or had WHO stage III (excluding pulmonary TB) or IV were offered ART. Blood samples for TLC and CD4 testing were collected in the same vacutainer tube containing EDTA at the study clinic and transported to the CDC laboratory in Entebbe. CD4 cell counts were measured by a dual-platform protocol using a FACScan instrument and Tritest reagents (Beckson-Dickenson, San Carlos, CA). TLCs were measured both using a hematology analyzer (Beckman-Coulter, Fullerton, CA) and the FACScan instrument. We have previously found that delays in transport of up to 5 days do not affect the accuracy of the FACScan results for CD4 cell counts (R. Downing, unpublished data) and therefore used the TLC results obtained on the FACScan in order to determine if transport time to the lab significantly affected the difference between the two methods. TLCs results from the hematology analyzer were used for the analysis. Clinical information during screening was collected using standardized instruments completed by study physicians. Subjects with WHO stage IV disease were excluded for this analysis.

Bivariate correlations between TLC results obtained on the FACscan and hematology analyzer were conducted. Differences between the two results were calculated and compared with results stratified by time between blood draw to testing of ≤ 1 day and > 1 day, using the Wilcoxan Rank Sum test. Distributions of TLCs, Hb and BMI across CD4 cell count strata were compared in a pair-wise fashion using the Wilcoxan Rank Sum test. Two by two tables were constructed to examine the association between different strata of TLCs, Hb and BMI with CD4 cell counts ≤ 200/μL or ≤350/μL. Sensitivity, specificity, positive and negative predictive values and accuracy (true positives + true negatives/all subjects) of the models were then calculated. We examined different TLC thresholds in terms of their ability to maximize sensitivity in detecting subjects with CD4 cell counts ≤ 200 cells/μL and minimize the proportion of subjects offered ART with cell counts > 350 cells/μL (false positives). Hb and BMI thresholds, alone and in combination, were then used to classify subjects with intermediate TLCs as qualifying for therapy. 95% confidence intervals for sensitivity and false positive ratios were calculated for the final models according to the Wald method [14]. Final models were then run under the scenario where all subjects with WHO stage III disease were offered ART and the algorithm was used to determine ART eligibility for those with stages I and II only. Final models were also examined separately for men and women.

The effects of tuberculosis, malaria parasitemia, or diarrhea at the time of screening were examined by conducting sub-group analyses which excluded subjects with these illnesses. All statistical analyses were conducted in SAS version 9.0 (SAS Institute, Cary, NC)

## Results

Between May 2003 and June 2005, 1944 HIV-infected adults presented for assessment of ART eligibility. Of these, 104 (5.2%) were excluded from this analysis as they were found to have WHO clinical stage IV disease. Another 53 individuals were excluded because of missing TLC or CD4 cell count values leaving 1787 subjects available for analysis. 75.2% were women and 27.8% were men. Median baseline CD4 cell counts and total lymphocyte counts, were 239 (inter-quartile range [IQR] = 119–411) and 1830 (IQR = 1420–2360) cells/μL, respectively. The mean time between blood sample collection and TLC and CD4 testing was 1.4 days; 56% of subjects had blood tested on the same day or one day after blood draw and 43% were tested 2 days after blood draw. One patient had blood tested 8 days after blood draw and was excluded from the analysis. TLC results between the hematology analyzer and the FACScan instrument were highly correlated (Pearson's r^2 ^= 0.85, p < 0.001) with the analyzer consistently giving greater values (median difference 383 cells/μL; IQR = 253 – 560). Differences were slightly greater for subjects whose blood samples were tested 2 days after blood draw in comparison with those whose blood was tested ≤ 1 day after draw (399 cells/μL vs. 360 cells/μL, p = 0.003).

In total, 763 (42.7%) subjects had baseline CD4 cell counts ≤ 200 cells/μL, 459(25.7%) had cell counts of 201–350 cells/μL and 565 (31.6%) had CD4 counts > 350 cells/μL (Table 1). Median baseline hemoglobin was 11.6 g/Dl (IQR = 10.3–12.8) and median baseline body mass index was 20.0 kg/m^2 ^(IQR = 18.-21.9). TLCs, Hb and BMI were distributed differently between the CD4 cell count strata (p < 0.01, for pair-wise comparisons for all parameters), with higher values for all parameters measured in the higher CD4 cell count strata.

**Table 1:** Distribution of total lymphocyte counts, hemoglobin and body mass index values by CD4 strata for 1787 HIV – infected subjects in Tororo, Uganda.

	**CD4 cell count ≤ 200 cells/μL** **(column 1)**	**P value (column 1 to 2)**	**CD4 cell count 200 – 350 cells/μL** **(column 2)**	**P value** **(column 2 to 3)**	**CD4 cell count > 350 cells/μL** **(column 3)**
Number of subjects (%)	763 (42.7)		459 (25.7)		565(31.6)
TLC – cells/μL median (interquartile range)	1460 (1120 – 1910)	<0.001	1880 (1570 – 2330)	<0.001	2330 (1910 – 2860)
Hb in g/dL median (IQR)	11.3 (10.0 – 12.4)	<0.001	11.8 (10.6 – 12.9)	<0.001	12.3 (11.4 – 13.2)
BMI in kg/m2 median (IQR)	19.7 (18.0 – 21.5)	<0.001	20.4 (18.9 – 21.9)	0.009	20.8 (19.2 – 22.8)

**Figure 1:**
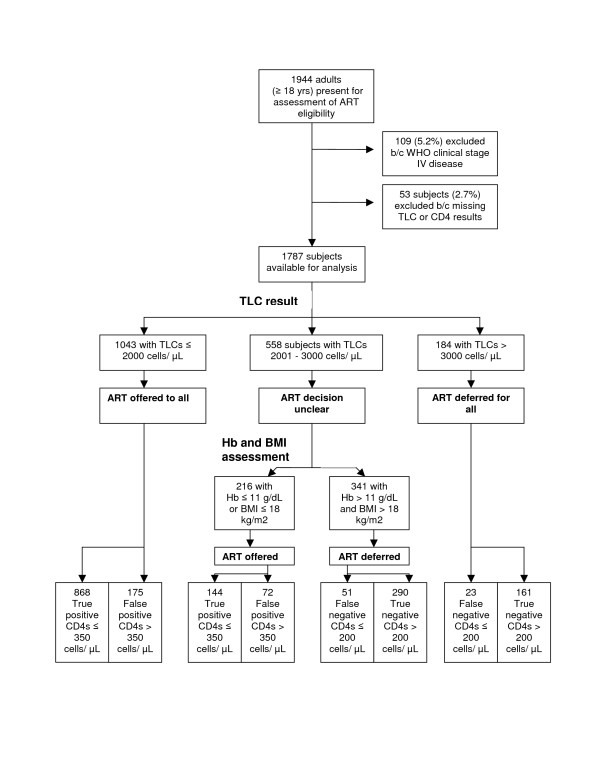
Algorithm for determining ART eligibility for 1787 HIV infected individuals using TLCs, Hb and BMI.

A TLC threshold of 2250 cells/μL was the most accurate (0.73) predictor of CD4 cell counts ≤ 350 cells/μL, yielding a sensitivity of 0.81 and a specificity of 0.54 (Table 2). This corresponded to a sensitivity of 0.88 (95% confidence interval [CI] 0.86 – 0.90) for CD4 cell counts ≤ 200 cells/μL and would result in 21% (95% CI 0.18 – 0.24) of subjects being offered ART with CD4 cell counts > 350 cells/μL (false positives). Incorporating Hb and/or BMI into algorithms produced two models with accuracy levels of 0.75. Both methods would offer ART to all subjects with TLCs ≤ 2000 cells/μL and defer treatment for those with TLCs > 3000 cells/μL. To evaluate subjects with TLCs between 2000 and 3000 cells/μL, the first model used Hb ≤ 11 g/Dl alone and had a sensitivity of 0.88 (95% CI 0.86 – 0.90) for CD4 cell counts ≤ 200 cells/μL and a false positive ratio of 0.18 (95% CI 0.15 – 0.21). The second model used Hb values ≤ 11 g/Dl or a BMI ≤ 18 kg/m2 to classify intermediate TLCs and resulted in a sensitivity of 0.90 (95% CI 0.88 – 0.92) and a false positive ratio of 0.20 (95% CI 0.17 – 0.23). Figure 1 describes the flow of patients through the second algorithm. The use of these algorithms would have resulted in treatment being offered to 1212 and 1268 subjects, respectively, in comparison to 1230 subjects, if all subjects with CD4 cell counts ≤ 350 cells/μL were offered treatment.

**Table 2:** Performance of algorithms using TLCs alone or in combination with Hb and BMI in predicting CD4 cell counts ≤ 350 cells/μL in the Home-Based AIDS Care project, Tororo, Uganda

**TLC Lower threshold**	**TLC Upper threshold**	**Hb threshold**	**BMI threshold**	**Sensitivity for CD4 <350**	**Specificity for CD4 <350**	**accuracy**	**sensitivity for CD4<200**	**False Positives**	**N Qualifying For ART**
A) CD4 alone				1.00	1.00	1.00	1.00	0	1230
B) TLC alone									
1500				0.40	0.95	0.67			519
1750				0.59	0.85	0.67	0.69	0.11	801
2000				0.71	0.69	0.71	0.80	0.17	1043
*2250				0.81	0.54	**0.73**	0.88	0.21	1250
2500				0.88	0.41	0.73	0.93	0.24	1404
2750				0.92	0.28	0.72	0.96	0.27	1523
3000				0.95	0.21	0.71	0.97	0.28	1601
									
C) TLC combined with Hb and/or BMI									
1750	2750	11		0.71	0.75	0.68	0.81	0.14	1017
1750	2750	12		0.78	0.62	0.73	0.86	0.18	1180
1750	2750		18	0.64	0.77	0.68	0.75	0.14	917
1750	2750	11	18	0.74	0.69	0.73	0.84	0.16	1087
*2000	3000	11		0.81	0.62	**0.75**	0.88	0.18	1212
2000	3000	12		0.85	0.51	0.74	0.94	0.21	1335
2000	3000		18	0.75	0.63	0.72	0.84	0.19	1138
**2000	3000	11	18	0.83	0.57	**0.75**	0.90	0.20	1268
1750	2500	11		0.69	0.76	0.72	0.79	0.14	989
1750	2500	12		0.79	0.61	0.73	0.84	0.20	1169
1750	2500	11	18	0.72	0.72	0.72	0.82	0.15	1045
2000	2750	11		0.79	0.62	0.74	0.86	0.18	1190
2000	2750		18	0.75	0.64	0.71	0.83	0.19	1128
2000	2750	11	18	0.81	0.57	0.74	0.89	0.20	1242
D) TLC combined with Hb and/or BMI for WHO stages I and II only									
**2000	3000	11		0.80	0.62	**0.74**	0.93	0.19	1282^‡^
2000	3000	11	18	0.83	0.57	**0.73**	0.90	0.20	1328^‡^
E) TLC combined with Hb and/or BMI for men only									
2000	3000	11		0.58	0.71	**0.72**	0.80	0.12	
2000	3000	12		0.76	0.63	0.73	0.84	0.14	
2000	3000	11	18	0.77	0.60	**0.73**	0.87	0.14	
**2000	3000	12	18	0.80	0.57	0.75	0.89	0.15	

Models with the highest accuracy using TLC alone (*) and TLC and Hb +/- BMI (**)

‡ Includes offering ART to 333 subjects with WHO stage III disease of whom 46 had CD4 cell counts > 350 cells/μL.

Results for women were unchanged from that of the whole group (data not shown). Both algorithms from the whole group analysis performed well for men separately, with accuracies of 0.72 and 0.73 for the models including TLC and Hb; and TLC, Hb and BMI, respectively (Table 2E). However, the highest accuracy was found using an algorithm with TLC thresholds of 2000 and 3000, to firstly include and exclude subjects for treatment; and Hb values of 12 g/dL and BMI of 18 kg/m2 to assign treatment to those with intermediate TLCs. This model had an accuracy of 0.75, a sensitivity for CD4 cell counts ≤ 200 cells/μL of 0.87 and a false positive ratio of 0.14.

Offering ART to all subjects with WHO stage III disease and using the algorithms to determine ART-eligibility for those with stage I or II disease would have resulted in treatment being offered to 1282 or 1328 subjects with similar levels of sensitivity and false positives. A total of 19 subjects had malaria parasitemia, 60 subjects were on TB treatment or diagnosed with TB at screening and 96 subjects had diarrhea at screening. However, excluding any of these subjects did not improve the predictive value of the final models.

## Discussion

We have demonstrated that using a TLC of 2250 cells/μL to determine ART eligibility in subjects with WHO clinical stages I, II or III, could identify 88% of subjects with CD4 cell counts ≤ 200 cells/μL, while 21% of subjects offered ART would have CD4 cell counts > 350 cells/μL. Using a TLC threshold of 2000 cells/μL to offer ART and an upper threshold of 3000 cells/μL to exclude subjects from treatment with Hb and BMI thresholds to determine ART eligibility for those with intermediate TLCs only marginally improved the accuracy of TLCs alone.

Despite the failure to improve accuracy of TLCs in predicting CD4 cell counts, using Hb and BMI may still be of value in determining who should initiate ART in resource-limited settings. Recent studies have shown that low Hb values and low BMI are independent risk factors for early mortality on ART in African settings [15,16]. Therefore incorporating Hb and BMI into ART eligibility criteria may help to prioritize treatment for those who are at increased risk of death if ART is delayed until CD4 cell counts drop further. ART guidelines in use in industrialized countries recommend treatment for individuals with CD4 cell counts in the range of 200 – 350 cells/μL primarily for those with factors which may limit the effectiveness of ART if treatment is much delayed [17]. The strategy proposed here adopts a similar approach but one which is likely more relevant to HIV-infected individuals living in sub-Saharan Africa.

The number of patients offered therapy under our TLC/Hb/BMI criteria were similar to the number offered treatment had ART been offered to all patients with CD4 cell counts ≤ 350 cells/μL, however, the same patients would not be treated under both scenarios. Between 17 and 19% of subjects with CD4 cell counts ≤350 cells/μL would not be offered ART using the TLC/Hb/BMI algorithms, however, as they would not have low BMIs or low Hb values, unless they also had TLCs >3000 cells/μL, it is unlikely that they would be at risk for early mortality without treatment.

More than 75% of the participants in this study were women. While the proportion of women aged 15 – 59 with HIV in Uganda is about 28% greater than men (7.3 versus 5.2%)[18], it is likely that differences in health seeking behavior are a more likely explanation for the large differences in the numbers of men and women seen in our study. When models were run excluding women from the analysis, we found that using a Hb threshold of 12 g/dL was a more accurate threshold for distinguishing which men had low CD4 cell counts.

If TLCs are to prove to be a viable alternative to using CD4 cell counting in managing HIV-infected individuals in resource-limited settings, they will need to be shown to be useful not only in determining when individuals should initiate ART, but also in monitoring patients on ART. There have been few studies on the use of TLCs in monitoring individuals on ART, but all have concluded that TLCs correlate well with changes in CD4 cell counts on ART[19,20]. However, the most recent ART guidelines from WHO state that "TLC is not suitable for monitoring therapy as a change in the TLC value does not reliably predict treatment success."[2] It is unclear from where this recommendation derives as no reference is provided.

The proportion of subjects in our study with CD4 cell counts ≤ 200 cells/μL and ≤ 350 cells/μL was very high (42.7% and 68.4%, respectively), resulting in a large number of subjects being ART-eligible. It is likely that our algorithm may not function as well in settings where the proportion of ART eligible subjects is not so high. However, uptake of voluntary counseling and testing is quite low in Uganda in that only 13% of women and 11% of men have ever been tested for HIV[18] and is also likely very low in other sub-Saharan African countries. Therefore it is reasonable to expect that many people presenting for assessment of ART-eligibility will have been tested for HIV because of ill health and a large proportion will likely meet eligibility criteria.

This study has two potential limitations. Firstly, TLCs and CD4 cell counts were not conducted at the site of blood drawing, but were transported to the CDC laboratory in Entebbe, which may have resulted in some deterioration of the blood samples. However, we had previously shown that CD4 cell count results from the FACScan instrument are stable up to 5 days after blood draw and the variability between the TLC values obtained on the FACScan were only a median of 40 cells/μL less for subjects whose blood was tested 2 days after draw compared to those whose samples were tested after ≤ 1 day delay between blood collection and testing. These observations suggest that transport time may not have caused significant inaccuracy in our laboratory test results. Secondly, the gold standard for determining inclusion or exclusion criteria for ART used in this study, that of CD4+ T lymphocyte counts, are also not perfect predictors of morbidity and mortality among people with HIV. While CD4 cell counting has proven useful in allowing stratification of risk for disease progression on ART individual variation of disease progression within CD4 cell count strata is large [9]. TLCs have been shown to be comparable predictors of mortality for HIV-infected populations receiving [21] and not receiving ART[22].

Thus, while TLCs may not correlate perfectly with CD4 cell counts, they may be as useful in predicting disease progression and therefore can be equally useful in determining when ART should be initiated. While evaluating how well ART-eligibility criteria based on TLCs perform in comparison to CD4 cell count – based criteria can only be answered by conducting clinical trials, this study has again demonstrated that TLCs are useful proxy measures for CD4 cell counts in determining ART eligibility.
